# Experimental and Numerical Analysis of the Quasi-Static and Dynamic Behavior of Silicate Materials

**DOI:** 10.3390/ma17235840

**Published:** 2024-11-28

**Authors:** Tomasz Jankowiak, Jakub Rafał Ossowski, Alexis Rusinek, Slim Bahi

**Affiliations:** 1 Institute of Structural Analysis, Poznan University of Technology, Piotrowo 5, 60-965 Poznan, Poland; 2 Silikaty Szlachta S.C., Ceramiczna 25, 83-243 Szlachta, Poland; jakub.ossowski.001@gmail.com; 3 Laboratory of Microstructure Studies and Mechanics of Materials (LEM3), ENSAM-Arts et Métiers ParisTech, UMR CNRS 7239, Lorraine University, 57078 Metz, France; alexis.rusinek@univ-lorraine.fr (A.R.); mohamed-slim.bahi@univ-lorraine.fr (S.B.)

**Keywords:** silicate, quasi-static, impact, CDP model, modeling, experiments

## Abstract

This study investigated both the static and dynamic behavior of silicate materials through a series of experimental and numerical tests. Compression tests were conducted on cubic samples, three-point bending tests on beams, and perforation tests on silicate plates. In the compression tests, stress–strain curves were generated, enabling the calibration of the Concrete Damaged Plasticity (CDP) model for silicate materials. The tensile strength of the silicate was assessed using three-point bending tests, while dynamic perforation tests determined the impact resistance of silicate when subjected to a rigid projectile. The perforation tests provided insight into the failure mechanisms of silicate plates under projectile impact at velocities approaching the ballistic limit. Additionally, the numerical simulations for all the experimental tests were performed using the Abaqus software in order to validate the accuracy of the material behavior model and confirm the appropriateness of the calibrated parameters for the chosen model. The results showed a strong qualitative and quantitative correlation with the experimental data, demonstrating the robustness of the adopted approach.

## 1. Introduction

Silicate blocks, particularly calcium silicate, are widely recognized as robust and versatile building materials in modern construction. These blocks are formed through an autoclaving process that involves a mixture of sand, lime (calcium oxide or calcium hydroxide), and water. The key chemical reaction in this process is hydration, which leads to the formation of calcium silicate hydrate [1], a material known for its high strength, durability, and resistance to environmental factors such as sulfate attack [2]. The hydration process imparts silicate blocks with advantageous properties such as high compressive strength, thermal insulation, and fire resistance, making them a preferred choice for load-bearing walls, partition walls, and other structural elements in residential, commercial, and industrial construction [3,4]. The performance of calcium silicate blocks (bricks) in masonry structures has been the subject of extensive research, particularly regarding their behavior under seismic loads. Studies indicate that while calcium silicate bricks possess advantageous properties, they can also be vulnerable to damage during seismic events, especially when used in unreinforced masonry buildings [5,6]. The anisotropic nature of masonry, which includes both bricks and mortar, complicates the assessment of structural performance under dynamic loads [2,7]. Furthermore, the moisture behavior of these bricks, particularly in cold climates, has been shown to significantly influence their hygrothermal performance, affecting the overall durability of masonry walls [8].

According to ecological rules with a growing trend toward circular construction, the research in article [9] shows that the use of up to 50% recycled calcium silicate brick aggregate in concrete and mortar maintains mechanical strength, increases frost resistance, and supports sustainable construction practices.

Calcium silicate blocks exhibit a variety of structural forms, including solid blocks, hollow blocks, and perforated blocks [10], each serving distinct roles depending on the construction requirements. These blocks are typically installed using traditional masonry techniques with mortar or adhesive and are often finished with plaster, paint, or external cladding materials [11,12]. Their composition and performance characteristics vary based on the specific manufacturing processes employed, yet they consistently demonstrate excellent resistance to moisture, pests, and decay, contributing to the longevity and integrity of the structures in which they are used [13].

A notable advancement in the silicate block category is autoclaved aerated concrete (AAC) blocks, which are lightweight and offer enhanced thermal insulation properties [14]. The unique porous structure of AAC blocks significantly reduces the weight of the material while maintaining sufficient strength for structural applications, thus providing both thermal and load-bearing advantages. These materials contribute to energy efficiency in buildings by improving insulation and reducing the need for active heating and cooling systems.

Research has shown that the mechanical performance of masonry made from calcium silicate blocks is influenced by factors such as joint thickness and mortar strength [15,16]. Studies reveal that thinner joints made with low-strength mortar significantly affect the mechanical properties of the masonry, while higher mortar strengths generally improve the overall structural performance. However, these studies often focus on the behavior of the walls as a whole rather than the mechanical properties of the individual blocks [17,18].

In contrast, this study aims to investigate the intrinsic mechanical properties of silicate blocks themselves, with a focus on compressive strength, tensile strength, and impact resistance. Unlike previous research, which predominantly examined static behaviors like compressive strength in wall assemblies, this study expands the scope to include both tensile strength and dynamic impact resistance of the individual blocks. This comprehensive mechanical analysis of silicate blocks is novel, combining experimental testing with numerical simulations to provide a holistic understanding of the material’s performance under various loading conditions. Such insights are crucial for optimizing the use of silicate materials in construction, particularly in applications requiring enhanced resilience to both static and dynamic stresses.

This research contributes to filling a gap in the literature by offering new experimental data and simulations that address not only the compressive behavior but also the tensile and impact properties of silicate blocks, thus broadening the understanding of their potential in construction [19,20].

## 2. Experimental Analysis and Description of the Geometry Specimens

The laboratory tests were conducted at the laboratory of the Institute of Structural Analysis, Poznan University of Technology. Compression tests were performed to characterize the strength and stress–strain behavior of the silicate material investigated in this study. Two silicate blocks, marked by the manufacturer (Silikaty Szlachta S.C., Szlachta, Poland) with the symbol N18A, were randomly selected from a single production batch [21]. The blocks were visually inspected for external defects, and their dimensions were carefully measured. The nominal dimensions of the blocks were 250 × 180 × 220 mm. The visual inspection and dimensional analysis were conducted in accordance with the PN-EN 771-2 standard [22] using calibrated measuring instruments.

**Figure 1:**
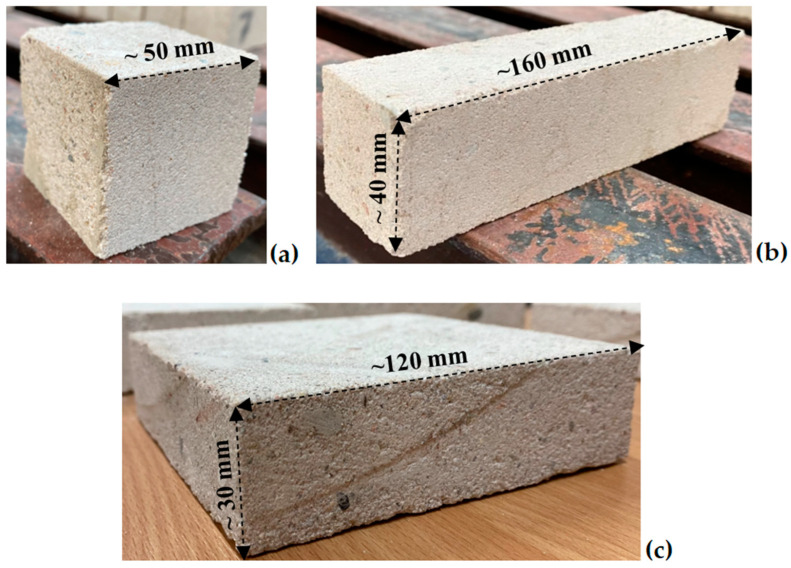
Selected silicate specimens for tests: (**a**) compression, (**b**) three-point bending, (**c**) perforation.

Subsequently, cubic samples with nominal dimensions of 50 × 50 × 50 mm were cut from the same large bricks. The cutting process was performed using a circular concrete saw with a diamond blade, ensuring accurate angles and precise sample dimensions. After discarding any damaged samples, a total of 23 samples were obtained, from which 12 were randomly selected for testing (see Table 1). A typical cubic sample is shown in Figure 1a [23].

The preparation of the samples ensured that the dimensions were consistent, with minimal dispersion during the cutting process (Table 1). Similarly, the mass of the samples showed limited variation. The dimensional measurements of the 23 specimens (length, width, and height) remained close to the nominal size of 50 mm, with average values of 50.7 mm, 50.6 mm, and 50.8 mm, respectively. Additionally, the average mass of the samples was 253.0 g, indicating a well-controlled preparation process. These results confirm that the samples were prepared with high precision, allowing for reliable testing and analysis; see the standard deviation of all quantities.

The three-point bending tests were conducted to evaluate the tensile behavior and strength of the silicate material. For this purpose, hollow elements marked by the manufacturer with the symbol N24PA, having nominal dimensions of 250 × 240 × 220 mm, were used to fabricate calcium silicate beams with nominal dimensions of 40 × 40 × 160 mm. This element belongs to the same strength class as the previously used N18A block and was sourced from the same material batch.

The three-point bending tests were conducted to evaluate the tensile behavior and strength of the silicate material. For this purpose, hollow elements marked by the manufacturer with the symbol N24PA, having nominal dimensions of 250 × 240 × 220 mm, were used to fabricate silicate beams with nominal dimensions of 40 × 40 × 160 mm. This element belongs to the same strength class as the previously used N18A block and was sourced from the same material batch. No cracks, chips, or other defects that could potentially affect the test results were observed. The average dimensions of the specimens, together with the standard deviations, are presented in Table 2. The same quantity is included for the mass of the specimen. The average dimensions are 161.5 mm × 41.6 mm × 41.4 mm, and the average mass is 541.6 g (Table 1).

Rectangular samples were cut using the same method described previously. A total of 15 samples were prepared, of which 2 were rejected due to imperfections and damage. An exemplary sample is depicted in Figure 1b. From the remaining 13 samples, 8 were randomly selected for further dimensional and mass measurements, with the results presented in Table 2 [23].

The perforation tests were conducted to assess the dynamic behavior of the calcium silicate material. The samples were composed of sand-lime blocks marked by the manufacturer as N24PA, which share the same strength class and originate from the same material batch as the N18A elements used in the static compression tests. Rectangular plates with nominal dimensions of 120 × 120 × 30 mm were cut using the same methodology applied to the previous silicate samples. The appearance of a representative sample is shown in Figure 1c.

Following the cutting process, 10 samples were obtained; however, 3 were discarded due to minor damage, such as visible cracks. Each remaining sample was meticulously dimensioned, with the final measurements presented in Table 3. The final inspection of the visual condition of the silicate samples confirmed the absence of defects and cracks that could adversely impact the macroscopic results of the material tests. The average dimensions of the specimens, together with the standard deviation, are presented in Table 2. The average dimensions are 122.3 mm × 121.9 mm × 33.5 mm, and the average mass is 911.8 g. The average density of the silicate material was determined to be 1600 kg/m^3^.

### 2.1. Definition of the Material Behavior Under Compression

**Figure 2:**
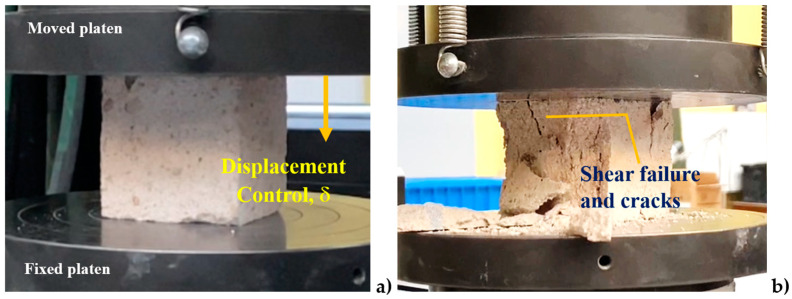
Experimental compression test: (**a**) sample prior to testing; (**b**) sample post-test displaying visible fracture mechanism.

An Instron hydraulic machine equipped with displacement control was used to conduct the compression tests on the silicate blocks, as shown in Figure 2a. During the experiments, the force, displacement, nominal stress, and strain were continuously recorded. The failure patterns were documented using a camera. Each test was terminated when the maximum force was reached and subsequently dropped by 40%. After attaining the maximum capacity, the samples were subjected to crushing. The maximum force $F_{max}$ and compressive strength $f_{c}$ for all samples are presented in Table 4, with the average compressive strength ${\bar{f}}_{c}$ calculated to be 15.2 MPa.

The typical failure pattern is illustrated in Figure 2b. During the tests, material softening and a drop in the stress–strain curve were observed. Additionally, the dispersion of the results, attributed to material anisotropy and heterogeneity, was quantified, yielding a standard deviation of ±1.9 MPa (12.6%).

**Figure 3:**
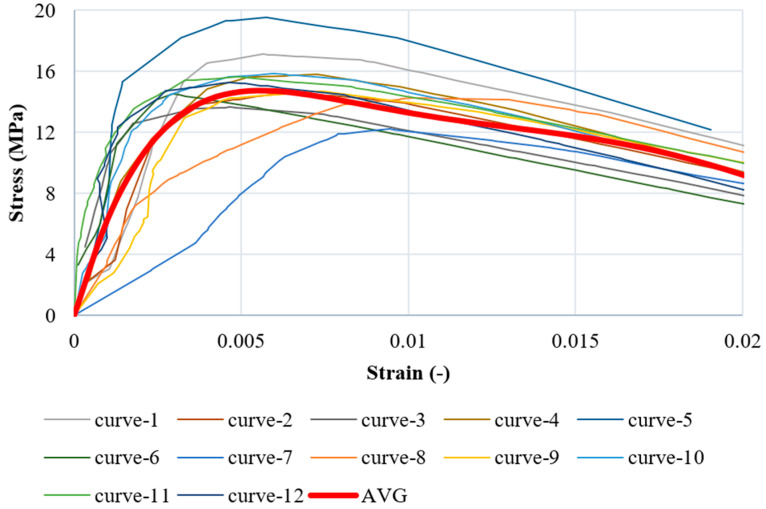
Stress–strain curves for compression of the silicate (experiments).

The compressive strength and maximum force values obtained for all tested specimens are detailed in Table 4. All results obtained from the experimental compression tests are presented in Figure 3. The average curve shown above serves as a basis for the calibration of the material model in the next sections.

A critical factor in the compression tests is the friction effect that occurs between the testing apparatus and the specimen [24,25,26]. Additionally, the shape of the sample influences the stress–strain curve. A comparison of the strengths obtained from cubic and cylindrical samples reveals that the compressive strength of cubic samples is approximately 15% higher, a trend commonly observed in typical concrete [23]. As a result, the actual material behavior is somewhat weaker than what was measured in the current tests [24,25,26]. This phenomenon is well documented in the international literature, primarily for ductile materials [26], but it also applies to brittle materials [24,25]. The friction in the compression of brittle materials reduces the displacement at the contact surface between the sample and the discs, and the complex stress state appears during compression. In the case of calcium silicate material, the general deformation of the specimen is close to 0.5% for the peak stress, and the effect of the Poisson ratio is visible. The effect of friction is the most visible in the softening phase of brittle materials [25].

### 2.2. The Three-Point Bending Test Description to Estimate the Silicate Strength

**Figure 4:**
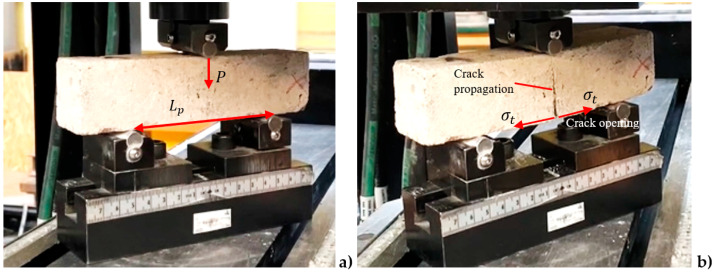
Experimental TPB test: (**a**) sample before the test; (**b**) sample after the test with visible fracture mechanism.

The same experimental setup (Instron machine) was employed to evaluate the bending capacity of the silicate beam using the three-point bending (TPB) method. This approach ultimately allows for the estimation of the tensile strength of the silicate material, denoted as $f_{t}$. The maximum bending forces recorded for all tests are summarized in Table 5, while a typical failure pattern is shown in Figure 4b, corresponding to the initial test configuration illustrated and discussed in Figure 4a.

Given the distance between the supports ($L_{p}$ = 100 mm), the maximum bending moment M was calculated using the formula $M=P\cdot L_{p}/4$, where P represents the applied load. To determine the maximum bending tensile stress $\sigma_{t}$, the maximum moment M is divided by the section modulus S. For a rectangular cross-section (approximately square in this analysis), the section modulus S is given by $W\cdot H^{2}/6$, as detailed in Table 2.

In the following figure, the sample can be seen before and after loading. The failure mode occurs through crack opening, corresponding to Mode I fracture (Figure 4b).

The average maximum bending force P recorded during the tests was 1315 N, while the average maximum tensile stress due to bending $\sigma_{t}$ was calculated to be 3.1 MPa with a standard deviation of ±0.29 MPa. However, the actual tensile strength $f_{t}\;$ is expected to be lower, as reported in the literature [27,28]. A detailed analysis explaining this discrepancy will be provided in Section 3.2.

### 2.3. Description of the Perforation and Impact Test

**Figure 5:**
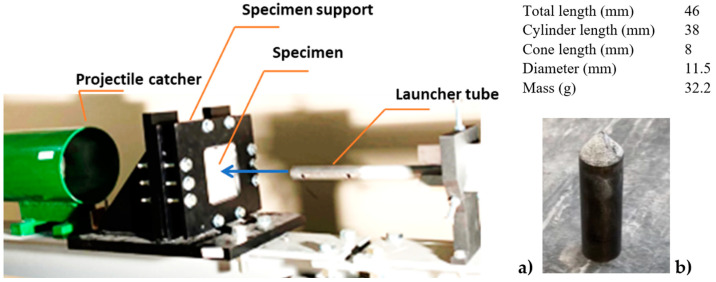
Experimental setup for perforation of the silicate plate: (**a**) sample fixed before the test; (**b**) conical projectile machined with hard steel.

The perforation tests demonstrated the impact resistance of the silicate material. Ballistics tests were conducted using a pneumatic gas gun that accelerates a conical projectile (Figure 5b) to reach an initial impact velocity $V_{0}$ at the end of the gas gun barrel (Figure 5a). The experimental setup for the perforation tests is presented, along with the system used to fix the sample. The sample holder ensures precise centering of the impact point and prevents any slipping during the test. The projectile, with its shape and characteristics, is also shown in Figure 5b.

Different initial velocities were achieved by varying the gas pressure in the tank, with typical values provided in Table 6 for pressures ranging from 2 to 5 bars. Using these initial velocities, the kinetic energy transferred to the specimens was calculated, with values approaching or below the ballistic limit. The considered range of initial velocities was between 44 m/s and 88 m/s, which corresponded to projectile kinetic energy from 31.2 J to 124.7 J. When $V_{0}$ exceeds the ballistic limit $V_{B}$, the projectile perforates the silicate plate, as observed in one of the tests.

**Figure 6:**
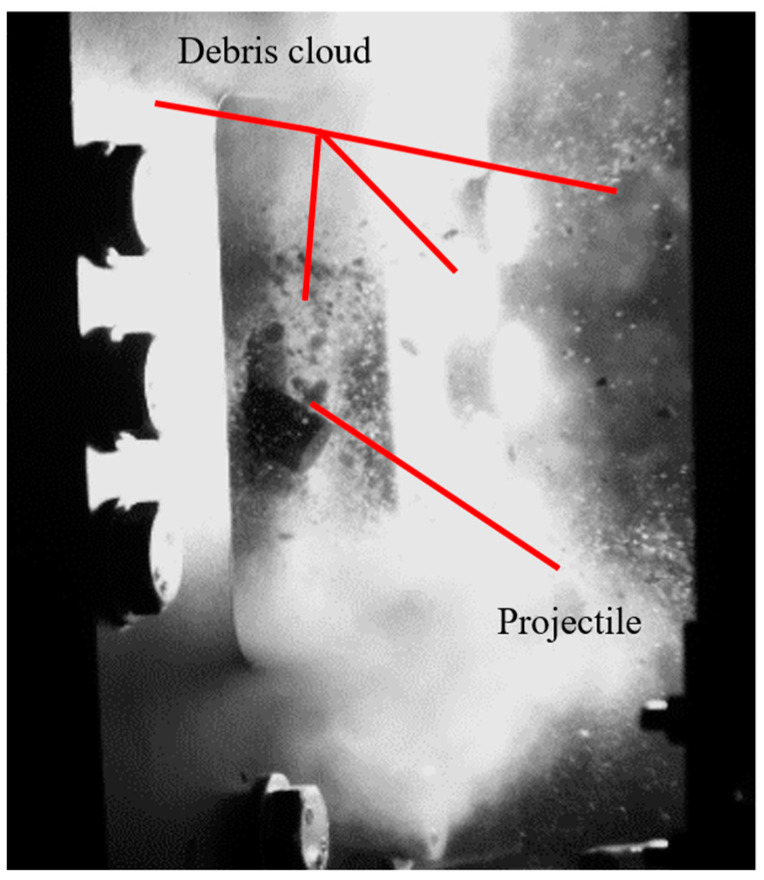
Recorded perforation of sample no. 1 (front view) for initial velocity of 88 m/s.

In one test, the projectile successfully perforated the sample at an initial velocity of 88 m/s, as shown in Figure 6. All detailed data and key results from the perforation tests are presented in Table 7, with additional information provided in Appendix A. Notably, perforation of the calcium silicate specimen occurred in 2 out of 3 tests when the maximum impact velocity was applied, as seen in Table 7 (tests no. 1 and 7).

**Figure 7:**
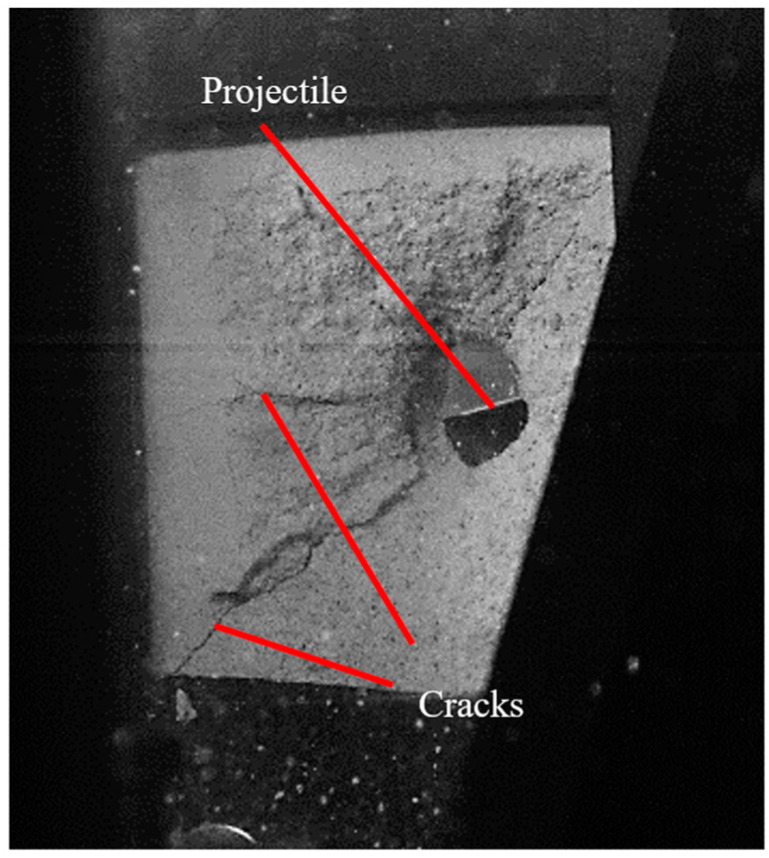
Frame during test no. 4 (the reflection of the projectile is visible—back view) for initial velocity of 88 m/s.

During test no. 4, the projectile was deflected at an initial velocity of 88 m/s (Figure 7). A residual velocity sensor, typically based on laser and mirrors as described in [29] for metal plates, was not used in this study due to the excessive amount of debris, which interfered with measurements. Instead, all tests were recorded using a high-speed camera with a time resolution of 20 μs, capturing both the front and back sides of the silicate plate.

At the lowest initial impact velocity, $V_{0}$ = 44 m/s (tests no. 3 and 6), no observable spalling was found on the back side, with only faint cracks visible. Consequently, no back hole diameter was observed. A small crater appeared on the front side, with a depth of 4 mm and a diameter of 5.8 mm in test no. 3 and 3.6 mm in depth and 9.5 mm in diameter in test no. 6. For the mid-range impact velocity of 67 m/s, perforation was incomplete. On the back side, the spalling diameters were 86 mm and 62 mm for tests no. 2 and 5, respectively. On the front side, a small crater with faintly visible cracks was observed, with diameters of 8.5 mm and 8.9 mm and depths of 8.2 mm and 7.6 mm, respectively. At the highest impact velocity tested, 88 m/s, spalling diameters of 83 mm, 84 mm, and 89 mm were produced in tests no. 1, 4, and 7, respectively. However, in test no. 4, the projectile failed to perforate the plate, instead rebounding and landing in front of the plate, as shown in Figure 7. Craters with exit holes were visible, with front side diameters of 18 mm, 19 mm, and 21 mm for tests no. 1, 4, and 7, respectively.

## 3. Numerical Simulations, Modeling of the Behavior, Damage, and Failure

The Abaqus/Explicit in Abaqus 2023 software [30] was employed to simulate all previously described experimental tests on the silicate blocks. In the simulations, silicate blocks were discretized using C3D8R solid finite elements. The Concrete Damage Plasticity (CDP) model was applied to represent the behavior of the silicate specimens, while other components, such as the compression plates, bending cylinders, and projectile used in the perforation test, were assumed to be rigid. General contact with a friction coefficient of 0.1 was implemented, and the simulations accounted for the creation of newly eroded surfaces in regions experiencing damage and failure under significant deformation. An accurate simulation of silicate damage and failure was essential, particularly for capturing behavior at advanced deformation stages. The CDP model, which has been widely used for concrete materials [30,31,32,33,34], was chosen here due to silicate’s mechanical similarity to concrete, including its softening behavior associated with micro-crack growth defined as “damage” in continuum mechanics. The model effectively differentiates between tension and compression behaviors, with softening predominantly occurring in the tensile zones where deformation localizes. This study applied the CDP model, as detailed in references [30,31,32,33,34]. The model integrates isotropic damaged elasticity with both tensile and compressive plasticity, capturing concrete-like irreversible behavior. It combines nonassociated multi-hardening plasticity and scalar (isotropic) damaged elasticity to model the progressive damage associated with fracture, allowing for element deletion based on material failure criteria. In the initial state, the CDP model [30,32,34] assumes isotropic elasticity for silicate, as specified in Appendix B (* Elastic keyword). As deformation progresses, the Cauchy stress tensor $\underline{\sigma }$, is defined relative to the effective stress tensor $\bar{\underline{\sigma }}$, as follows:

(1) $$\underline{\sigma }=\left(1-d\right)\;\bar{\underline{\sigma }}\;,$$

where d is a scalar damage variable (0≤d≤1). The model uses typical additive strain rate decomposition, which is valid for small elastic strain:

(2) $$\dot{\underline{\varepsilon }}={\dot{\underline{\varepsilon }}}^{el}+{\dot{\underline{\varepsilon }}}^{pl},$$

where $\dot{\underline{\varepsilon }}$ is total strain rate tensor, ${\dot{\underline{\varepsilon }}}^{el}$ is elastic and ${\dot{\underline{\varepsilon }}}^{pl}$ is plastic part of the strain rate tensor. The effective stress tensor $\underline{\sigma }$ is related to the strain difference $\underline{\varepsilon }-{\underline{\varepsilon }}^{pl}$:

(3) $$\bar{\underline{\sigma }}=D_{0}^{el}\;\left(\underline{\varepsilon }-{\underline{\varepsilon }}^{pl}\right)\;,$$

where $D_{0}^{el}$ is initial undamaged elastic stiffness of the material. The degraded elastic stiffness $D^{el}$ is equal to $\left(1-d\right)\;D_{0}^{el}$. The evolution of the scalar damage variable d is the following:

(4) $$d=d\left(\bar{\underline{\sigma }},{\tilde{\varepsilon }}^{pl}\right)\;,$$

where ${\tilde{\varepsilon }}^{pl}$ is a set of strain hardening (softening) variables:

(5) $${\tilde{\varepsilon }}^{pl}=\left[\begin{array}{c} {\tilde{\varepsilon }}_{t}^{pl}\\ {\tilde{\varepsilon }}_{c}^{pl} \end{array}\right]\mathrm{and}\;{\dot{\tilde{\varepsilon }}}^{pl}=h\left(\bar{\underline{\sigma }},{\tilde{\varepsilon }}^{pl}\right)\cdot {\dot{\underline{\varepsilon }}}^{pl}.$$

The strain hardening and softening of the material in compression and in tension are calibrated based on the uniaxial compressive test (point 2.1) and based on the tripoint bending test (point 2.2). The initial stiffness of the material is calculated based on the average stress–strain curve (see Figure 3). All the material parameters used in the simulations are presented in Appendix B.

The emergence of microcracks and concrete crushing is indicated by the progressive evolution of the hardening variables. These variables govern the development of the yield surface $F\left(\bar{\underline{\sigma }},{\tilde{\varepsilon }}^{pl}\right)$ and the degradation of the elastic stiffness. Additionally, they are closely connected to the dissipated fracture energy necessary for the initiation of microcracks. The yield function, $F\left(\bar{\underline{\sigma }},{\tilde{\varepsilon }}^{pl}\right)\leq 0$, defines a surface within the effective stress space, and it plays a critical role in determining failure or damage states. The yield function proposed by Lubliner et al. [34] is used in the CDP model as follows:

(6) $$F\left(\bar{\underline{\sigma }},{\tilde{\varepsilon }}^{pl}\right)=\frac{1}{1-\alpha }\left(\bar{q}-3\alpha \bar{p}+\beta \left({\tilde{\varepsilon }}^{pl}\right){\bar{\sigma }}_{max}-\gamma -{\bar{\sigma }}_{max}\right)-{\bar{\sigma }}_{c}\left({\tilde{\varepsilon }}_{c}^{pl}\right)\leq 0.$$

The procedures of the parameters α, γ, and function $\beta \left({\tilde{\varepsilon }}^{pl}\right)$ identification are explained in [32]. The variables $\bar{p}$, $\bar{q}$ and ${\bar{\sigma }}_{max}$ are effective hydrostatic pressure, Mises equivalent effective stress, and maximum effective stress. The Macauley bracket 〈·〉 in the previous formula makes γ parameter meaningful only in the triaxial compression state of stress if ${\bar{\sigma }}_{max}\geq 0$ in the meridian plane. The biaxial compression strength affects the parameter α. The function ${\bar{\sigma }}_{c}\left({\tilde{\varepsilon }}_{c}^{pl}\right)$ describes the uniaxial compressive behavior (see keyword * Concrete Compression Hardening). In the CDP model, the nonassociated plastic flow rule is used as follows:

(7) $${\dot{\underline{\varepsilon }}}^{pl}=\dot{\lambda }\frac{\partial G\left(\bar{\underline{\sigma }}\right)}{\partial \bar{\underline{\sigma }}}\;,$$

where

(8) $$G=\sqrt{{\left(\epsilon \;\sigma_{t0}\tan\psi \right)}^{2}+{\bar{q}}^{2}}-\bar{p}\tan\psi \;.$$

**Figure 8:**
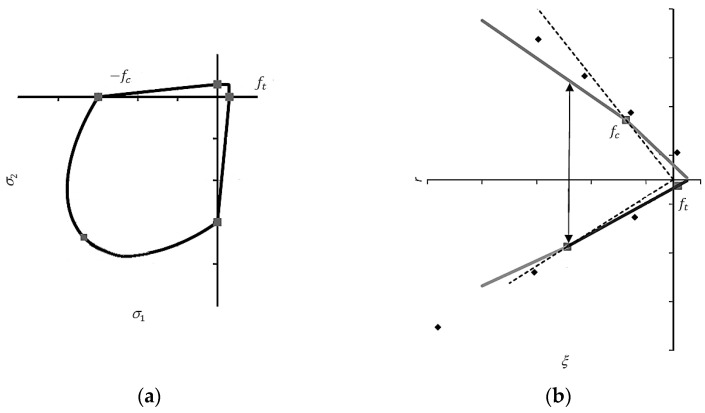
The loading function *F* in (**a**) plane stress condition and (**b**) meridian plane [32].

In the previous relation, Equation (8), ψ is the dilation angle in the meridian plane $\bar{p}-\bar{q}$ for high triaxial compression and $\sigma_{t0}$ is uniaxial tensile stress at failure. ϵ is the eccentricity of the plastic flow potential. Further research should be focused on the experimental test in an advanced state of stress; however, only the compressive and tensile zones were investigated in this research (compression and 3-point bending test). The parameters of the yield function F and of the plastic potential G in the simulations are defined in Appendix B (see keyword * Concrete Damaged Plasticity). In the present paper, the typical values of these four parameters for concrete are assumed due to the lack of the triaxial test of the silicate. To clarify the effect of these parameters, the loading function F is presented in Figure 8 in plane stress and in the meridian plane. Based on the tests presented in Section 2.1 and Section 2.2, the evolution of the internal variables, such as equivalent plastic strain and damage variables in tension and compression, is performed. In CDP, the fracture energy regularization of the material model in the tensile zone is used. The softening part of the stress–strain curve in tension is represented in the model by the stress–displacement curve. The linear relation is used in the approximation (see keyword * Concrete Tension Stiffening). The appropriate damage curves in compression and tension are also presented (see keywords * Concrete Compression Damage and * Concrete Tension Damage in Appendix B).

In summary, the CDP model in Abaqus incorporates three main considerations for modeling the behavior of silicate material: (1) an asymmetry in yield strength between tension and compression, (2) an initial phase of hardening followed by softening under compressive loads, alongside purely softening behavior under tensile loads, and (3) differential degradation of elastic stiffness in tension versus compression. For all simulations, a consistent mesh discretization was applied with a characteristic finite element length of 2 mm to ensure uniform accuracy across analyses. The authors checked the effect of the mesh size on the numerical results, and the pathological mesh size dependency was not observed due to regularization. The subsequent sections present the principal results from the numerical simulations for compression, tension, and perforation, each compared with the experimental outcomes to validate the model’s accuracy.

### 3.1. Compression Test of Silicate, Comparison Between Experiments, and Modeling

**Figure 9:**
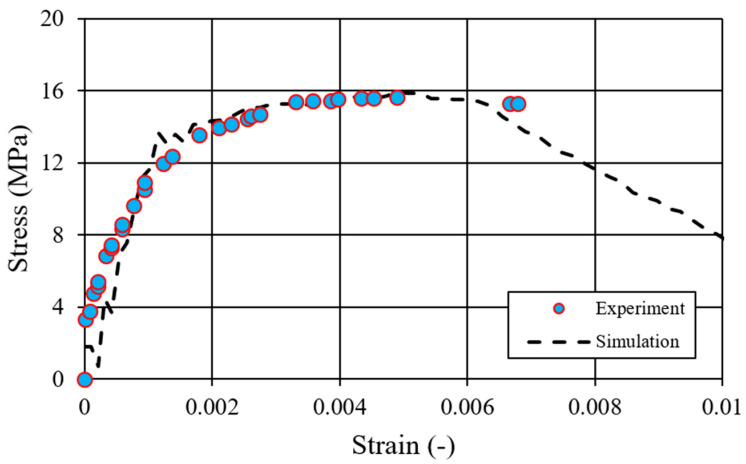
Comparison of stress–strain curves in compression: experimental data vs. simulation.

**Figure 10:**
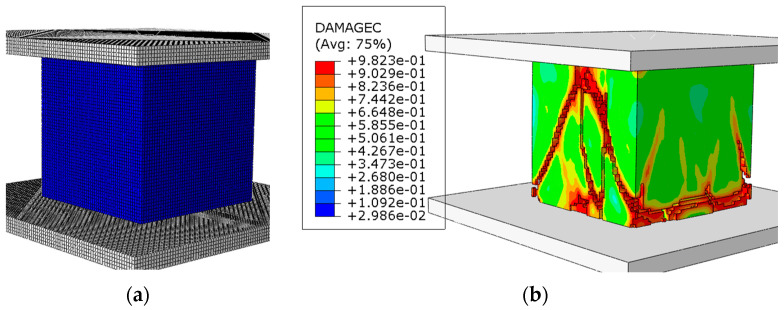
Numerical model: (**a**) sample before the test (mesh); (**b**) sample after the test with visible fracture mechanism.

The stress–strain curves derived from numerical simulations were compared to those recorded during experimental testing, as illustrated in Figure 9. These curves show a good agreement, indicating that the simulation accurately reflects the material behavior under compressive load. The simulation results demonstrate the expected response characteristics of the silicate material, including the initial elastic region, the yield point, and subsequent softening behavior. This agreement confirms the accuracy of the CDP model in representing the progressive material damage and failure modes observed experimentally. Additionally, the failure pattern observed in the simulations, described in Figure 10, is similar to the one observed during experiments, showing a similar shear-dominant damage mechanism and crack propagation pattern. The development of shear bands and the gradual coalescence of microcracks into primary failure planes can be seen both in the simulated and experimental results. This shear-dominated asymmetric failure is typical for brittle materials under compressive loading, where localized shear zones precede the ultimate collapse.

Moreover, the model prediction of post-failure behavior captures the subtle changes in stress redistribution within the material, further supporting its validity. This agreement between the simulated and experimental failure patterns suggests the CDP model’s robustness in capturing not only peak strength but also the softening and residual stress response of the silicate material under load for friction coefficient 0.1. The basic limitation of using the CDP model is that it is isotropic at the beginning and starts to be anisotropic after the first plasticity or damage.

### 3.2. Three-Point Bending Test, Comparison Between Experiments, and Simulations

**Figure 11:**
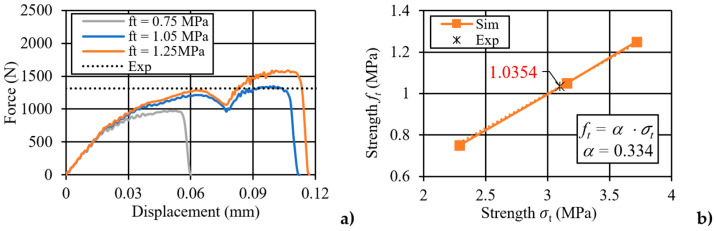
Numerical analysis results: (**a**) force–displacement curve; (**b**) strength *f_t_* versus strength $\sigma_{t}$ trend (calibration with experiment).

The force–displacement curves for three numerical cases, each with a different assigned tensile strength ($f_{t}$ equal to 0.75 MPa, 1.05 MPa, and 1.25 MPa), are displayed in Figure 11a. For comparison, the experimentally obtained average maximum force value is also presented in the same figure. The curves reveal how variations in tensile strength impact the force–displacement response of the silicate material, providing insights into the model’s sensitivity to tensile strength adjustments. In Figure 11b, the relationship between tensile strength $f_{t}$ and bending strength $\sigma_{t}\;$ is shown as a linear trend, with a slope value of α equal to 0.334, which is applied as a correction factor to refine the model’s tensile strength parameters. This linear correlation indicates that as tensile strength increases, the corresponding bending strength follows predictably, supporting the use of this slope as a reliable scaling factor. The established correction factor enables an accurate calibration of tensile strength within the model, enhancing its predictive capabilities under bending conditions. Additionally, the use of notched silicate specimens subjected to three-point bending tests is noted, as these configurations are occasionally employed to study fracture mechanics in brittle materials.

**Figure 12:**
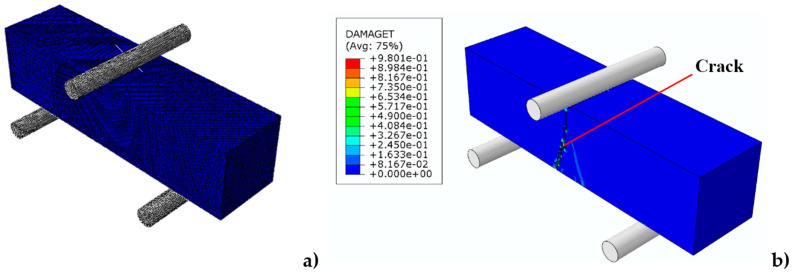
Numerical model results: (**a**) sample before the test; (**b**) sample after the test with visible fracture mechanism.

In such cases, crack initiation and propagation generally occur along the vertical axis, corresponding to the direction of maximum tensile stress, as observed in previous studies [31]. This calibrated model, with adjusted tensile properties, is expected to accurately capture the initiation and progression of fractures, especially in scenarios involving tensile and bending stress distributions (Figure 12).

The calibration process outlined indicates that the tensile strength of the silicate material $f_{t}$ has been determined to be 1.05 MPa. This calibrated value plays a crucial role in the accuracy of numerical simulations related to both compression and perforation tests. By incorporating this tensile strength into the numerical models, it is ensured that the material response to loading conditions reflects realistic behavior, thereby being able to predict the validities of the simulations. In the context of the compression tests, the calibrated tensile strength is essential for understanding the interplay between tensile and compressive stresses during loading. Similarly, for the perforation tests, accurate tensile strength values are important for modeling the material resistance to impact and penetration, which can significantly influence the failure mechanisms observed during experiments.

### 3.3. Perforation Tests, Validation of the Numerical Model, Comparison Between Experiments, and Simulations

The numerical model for perforation is defined by a silicate plate represented by the Concrete Damage Plasticity (CDP) material model, while the projectile is assumed to be a rigid body. The fixation systems consist of two square rigid frames that secure the plates in place. The perforation models are developed based on the experimental configurations detailed in Section 2.3. Three initial impact velocities were considered in the simulations. The failure patterns observed during the tests are summarized in Table 8. Key parameters, including the front hole diameters, front hole depths, and back hole diameters, were compared between experimental and numerical results. The numerical and experimental data show a strong agreement. The projectile successfully perforated the silicate plate only at an initial impact velocity of 88 m/s, while the ballistic limit was estimated to be approximately 67 m/s.

**Figure 13:**
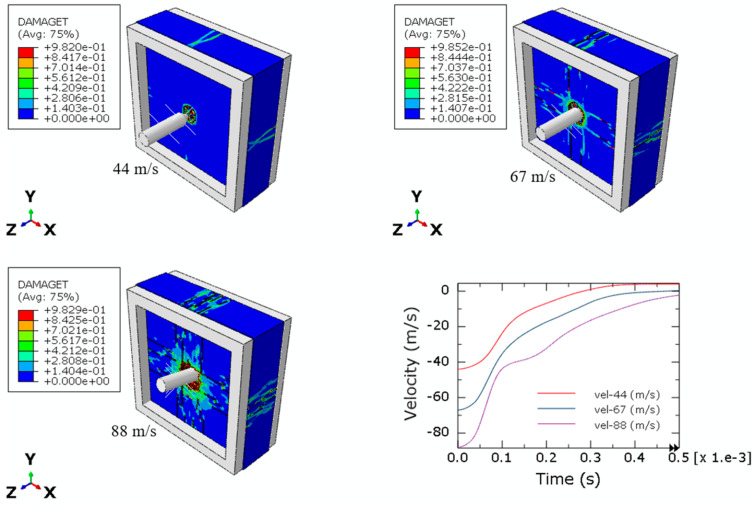
Perforation numerical results for initial velocities 44 m/s, 67 m/s, and 88 m/s (front view).

**Figure 14:**
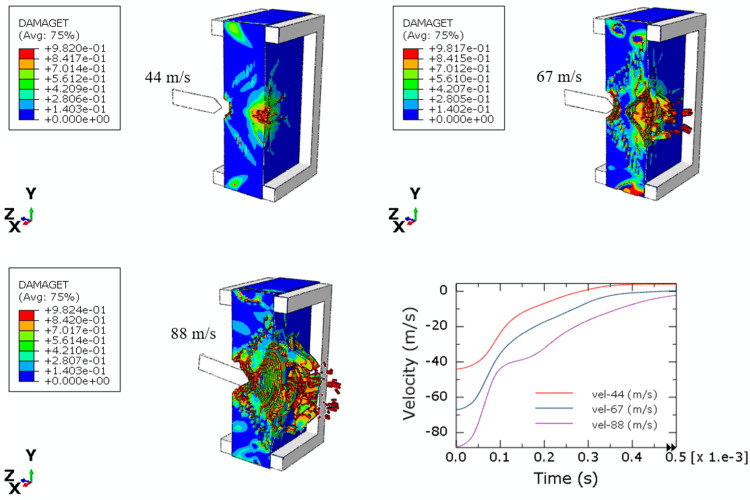
Perforation numerical results for initial velocities 44 m/s, 67 m/s, and 88 m/s (back view with cut plane).

Similar observations were noted in the numerical simulations, as illustrated in Table 8 and Figure 13 and Figure 14. The damage and failure patterns for all three impact simulations (with initial velocities of 44 m/s, 67 m/s, and 88 m/s) agree with those observed during experimental tests, both in front and back views. This consistency between experimental and numerical reinforces the validity of the numerical model in predicting the behavior of silicate materials under perforation conditions.

Numerical simulations were employed to define the projectile velocity over time, as illustrated in Figure 13 and Figure 14. The projectile experiences deceleration upon contact with the silicate plate. Notably, perforation of the plate occurs only at the maximum impact velocity. The spalling failure mode, characterized by a cup–cone formation, is observed in the simulations, mirroring the behavior seen during the experimental tests. Furthermore, the extent of damage to the silicate material is directly influenced by the initial impact velocity of the projectile, indicating a clear correlation between impact velocity and the resulting failure mechanisms.

## 4. Conclusions

The present study investigated the behavior of silicate material under both static and dynamic loading conditions. Experimental compression tests were conducted on cubic samples, while the tensile strength of the silicate was evaluated through three-point bending tests. In addition, the resistance of silicate to dynamic impacts with a rigid projectile at velocities near the ballistic limit was assessed. The experimental findings facilitated the calibration of a numerical model that is commonly employed to simulate the behavior of brittle materials. A critical aspect of accurately representing the experimentally observed behavior in numerical simulations was the selection of the appropriate material model. In this work, the Concrete Damage Plasticity (CDP) model was used for all simulations. This model is particularly suitable for brittle materials, as it effectively captures the non-linear and asymmetric (in compression and in tension) responses typical of such materials under varying stress states. The computational analyses encompassed all three types of experiments: compression, three-point bending, and perforation. The results obtained from the numerical simulations were in correct agreement with the experiments, both quantitatively and qualitatively, demonstrating the robustness of the CDP model in representing the mechanical behavior of silicate.

The most significant contributions of this study to the understanding of calcium silicate brick material are as follows:

- Original loading tests for various strain rates and loading paths: The research incorporated a comprehensive range of loading scenarios, including compression, three-point bending, and impact tests. This variety is instrumental in the calibration of constitutive models, providing a more nuanced understanding of the behavior of materials under different loading conditions;
- Calibration of the numerical model for the analyzed calcium silicate: The successful calibration of the CDP model specifically for silicate enables more accurate predictions of material performance in practical applications, which is essential for engineering and structural design;
- Conducting numerical simulations of all experimental tests: The integration of numerical simulations with experimental data allows for a thorough validation of the model considering material behavior and boundary conditions. The presented research helps to design more reliable structures made of calcium silicate bricks;
- Validation of the model through comparative analysis: The validation process, which involves the comparison of numerical results with experimental data, provides confidence in the applicability of the model for future studies and practical implementations. This aspect is crucial to advance the understanding of brittle materials and optimize their use in structural applications.

Therefore, this research not only improves the understanding of calcium silicate materials under various loading conditions but also establishes a reliable numerical framework to predict their behavior, thus contributing to the broader field of material science and engineering.

The limitations of the laboratory tests presented do not include the tests of pure compression and tension dynamic behavior. These tests allow for the exploration of the strain rate sensitivity of the calcium silicate material. Additionally, impacts at higher velocities could be simulated. Another limitation is the isotropy of the virgin material model, which becomes anisotropic during deformation due to plasticity or damage.

## Figures and Tables

**Table 1:** Dimensions of the selected compressive samples.

No. Sample	Dimensions (mm)			Mass (g)
	L (Length)	W (Width)	H (Height)	
1	50.1	50.0	50.6	246.0
2	50.5	50.9	50.5	248.2
3	51.2	50.3	51.1	257.2
4	50.6	50.4	50.8	245.8
5	50.9	50.5	50.8	247.8
6	51.2	51.1	51.1	270.8
7	50.5	50.2	50.8	250.0
8	51.0	51.3	51.2	279.6
9	50.4	50.5	51.0	259.0
10	50.2	50.2	50.0	245.8
11	51.1	51.0	50.8	243.0
12	51.0	50.5	50.8	243.0
*Average value*	50.7 mm	50.6 mm	50.8 mm	253.0 g
*Standard deviation*	±0.372 mm	±0.390 mm	±0.309 mm	±11.14 g

**Table 2:** Dimensions of the selected three-point bending samples.

No. Sample	Dimensions (mm)			Mass (g)
	L (Length)	W (Width)	H (Height)	
1	164.0	42.0	41.0	533.8
2	161.0	41.0	42.0	534.2
3	161.0	42.0	41.0	534.2
4	161.0	42.0	41.0	529.6
5	161.0	41.0	42.0	551.0
6	161.0	41.0	42.0	571.2
7	161.0	43.0	41.0	551.6
8	162.0	41.0	41.0	527.2
*Average value*	161.5 mm	41.6 mm	41.4 mm	541.6 g
*Standard deviation*	±1.000 mm	±0.696 mm	±0.484 mm	±14.08 g

**Table 3:** Dimensions of the selected impacted samples.

No. Sample	Dimensions (mm)			Mass (g)
	L (Length)	W (Width)	T (Thickness)	
1	121.5	121.0	33.0	901.2
2	121.8	126.0	32.0	904.6
3	121.6	121.0	34.0	910.1
4	121.0	121.0	34.0	907.8
5	127.1	122.0	34.5	924.2
6	121.0	121.0	32.0	913.8
7	122.0	121.0	35.0	921.2
*Average value*	122.3 mm	121.9 mm	33.5 mm	911.8 g
*Standard deviation*	±1.996 mm	±1.726 mm	±1.102 mm	±7.83 g

**Table 4:** The compressive strength.

No. Sample	Maximum Force (N)	Compressive Strength (MPa)	No. Sample	Maximum Force (N)	Compressive Strength (MPa)
1	42,823	17.1	7	31,309	12.2
2	36,436	14.6	8	37,548	14.4
3	34,062	13.6	9	37,315	14.7
4	39,674	15.9	10	39,582	15.8
5	48,951	19.6	11	40,706	15.6
6	36,394	14.6	12	38,417	15.2
*Average value of the compressive strength*					15.2 MPa
*Standard deviation of the compressive strength*					±1.9 MPa

**Table 5:** The bending capacity.

No. Sample	Maximum Bending Force (N)-*P*	Maximum Tensile Stress Due to Bending (MPa)-*σ_t_*
1	1150	2.69
2	1290	3.01
3	1450	3.41
4	1350	3.16
5	1090	2.55
6	1400	3.27
7	1370	3.21
8	1420	3.32
*Average value*	1315 N	3.1 MPa
*Standard deviation*	±122.1 N	±0.29 MPa

**Table 6:** The shooting parameters of the gas gun.

Gas Gun Pressure (bar)	Initial Impact Velocity (m/s)	Projectile Kinetic Energy (j)
2	44.0	31.2
3	67.0	72.3
5	88.0	124.7

The color corresponds with the impact velocity: darker grey color means higher speed.

**Table 7:** Failure pattern description for all tests.

No. Sample	Initial Velocity (m/s)	Front Hole Diameter (mm)	Front Hole Deep (mm)	Back Hole Diameter (mm)
1	88	18.0	Exit hole (perforation)	83.0
2	67	8.5	8.2 (reflection)	86.0
3	44	5.8	4.0 (reflection)	Lack
4	88	19.0	Exit hole (reflection)	84.0
5	67	8.9	7.6 (reflection)	62.0
6	44	9.5	3.6 (reflection)	Lack
7	88	21.0	Exit hole (perforation)	89.0

The color corresponds with the impact velocity: darker grey color means higher speed.

**Table 8:** Failure pattern description for all simulations versus experiments.

Initial Velocity (m/s)	Front Hole Diameter (mm)		Front Hole Deep (mm)		Back Hole Diameter (mm)	
	Experiment	Simulation	Experiment	Simulation	Experiment	Simulation
88	19.3	20	Exit hole (perforation)	Exit hole (perforation)	85.3	88.0
67	8.7	12	7.9 (reflection)	9 (reflection)	74.0	60.0
44	7.7	10	3.8 (reflection)	6 (reflection)	Lack	Lack

The color corresponds with the impact velocity: darker grey color means higher speed.

## Data Availability

The original contributions presented in this study are included in the article. Further inquiries can be directed to the corresponding author.

## Appendix A

The following failure mechanisms in perforation tests are obtained. All data compatible with Table 7 for the maximal velocity are presented in Table A1. The same information for the middle velocity is presented in Table A2 and Table A3 for the lowest impact velocities.

**Table A1:** Perforation results for initial velocity of 88 m/s.

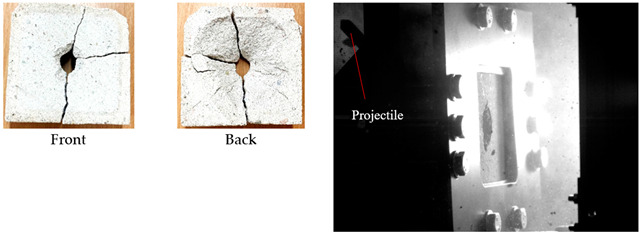

**Test no. 1** (shooting pressure 5 bars, initial velocities 88 m/s) The cracks open after removing the plate from the support system. Both side views are presented, as well as the frame from the recording with a visible hole and projectile after perforation (which turns).

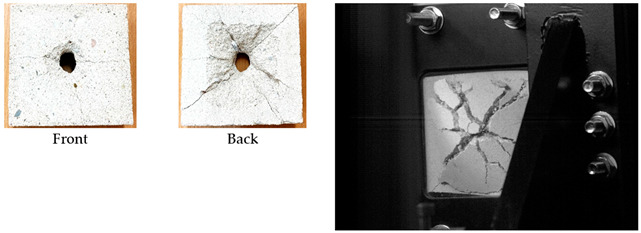

**Test no. 4** (shooting pressure 5 bars, initial velocities 88 m/s) The cracks are not visible on the front side. Both side views are presented, as well as the frame from the recording with visible spalling.

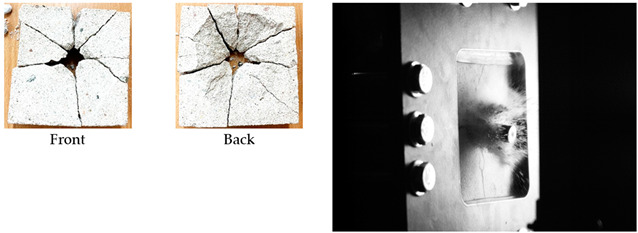

**Test no. 7** (shooting pressure 5 bars, initial velocities 88 m/s) The cracks on the front side open during impact. Both side views are presented, as well as the frame from the recording with a visible hole and the projectile that perforated the plate.

**Table A2:** Perforation results for initial velocity of 67 m/s.

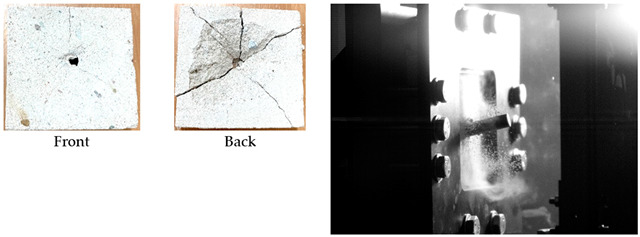

**Test no. 2** (shooting pressure 3 bars, initial velocities 67 m/s) The cracks are barely visible on the front side, and it opens after removing the plate from the support system. Both side views are presented, as well as the frame from the recording with a visible crater and projectile.

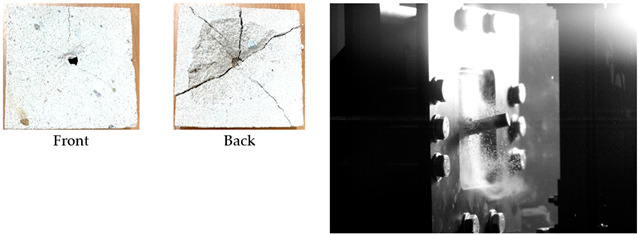

**Test no. 5** (shooting pressure 3 bars, initial velocities 67 m/s) The cracks are barely visible on the front side. Both side views are presented, as well as the frame from the recording with a visible spalling.

**Table A3:** Perforation results for initial velocity of 44 m/s.

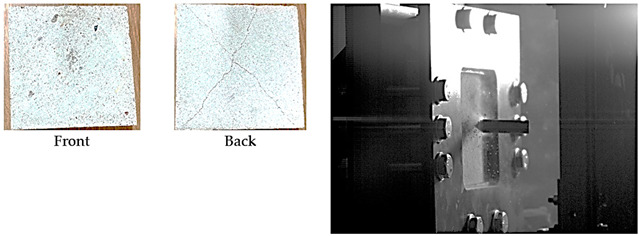

**Test no. 3** (shooting pressure 2 bars, initial velocities 44 m/s) The cracks are not visible on the front side and are barely visible on the end side. Both side views are presented, as well as the frame from the recording with a small visible crater and projectile.

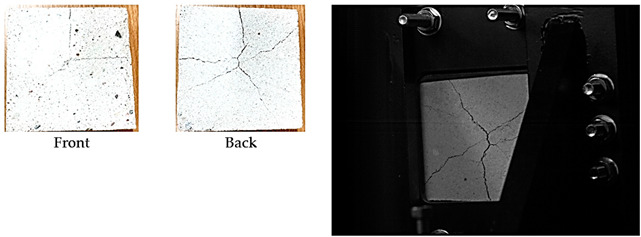

**Test no. 6** (shooting pressure 2 bars, initial velocities 44 m/s) The cracks are not visible on the front side and are barely visible on the end side. Both side views are presented, as well as the frame from the recording with visible cracks during impact.

## Appendix B

The complete definition of the silicate material used in the presented simulations is in Table A4.

**Table A4:** Material definition (CDP model in SI units for Abaqus input file and * are keywords).

* Density 1600, * Elastic 5.2e9., 0.2 * Concrete Damaged Plasticity 36., 0.1, 1.15, 0.666 * Concrete Compression Hardening 11.906e6, 0. 12.385e6, 0.00039 12.859e6, 0.0008697 13.333e6, 0.0014491 14.255e6, 0.0032456 14.574e6, 0.0051084 8.19e6, 0.1 * Concrete Compression Damage 0., 0. 0., 0.00039 0., 0.0008697 0., 0.0014491 0., 0.0032456 0., 0.0051084 0.43804, 0.1 * Concrete Tension Stiffening, type = displacement 1.25e6, 0. 0.0125e6, 0.15e-3 * Concrete Tension Damage, type = displacement 0., 0. 0.99, 0.15e-3 * Concrete Failure, type = displacement , , 0.98,
